# Effects of Jie Yu Wan on Generalized Anxiety Disorder: A Randomized Clinical Trial

**DOI:** 10.1155/2022/9951693

**Published:** 2022-04-08

**Authors:** Xue Li, Sisi Zheng, Sitong Feng, Rui Ma, Yuan Jia, Anquan Zhao, Dan Wei, Hua Guo, Na Duan, Ying Ding, Jindong Chen, Hong Zhu, Hongxiao Jia

**Affiliations:** ^1^The National Clinical Research Center for Mental Disorders & Beijing Key Laboratory of Mental Disorders & Beijing Anding Hospital, Capital Medical University, Beijing 100088, China; ^2^Tangshan Fifth Hospital, Tangshan, Hebei 063005, China; ^3^Zhumadian Mental Hospital, Zhumadian, Henan 463099, China; ^4^Hangzhou Seventh People's Hospital, Hangzhou, Zhejiang 310013, China; ^5^Xiamen Xian Yue Hospital, Xiamen, Fujian 361012, China

## Abstract

**Objective:**

To systematically assess the clinical efficacy of the *Jie Yu Wan* (JYW) formula in treating generalized anxiety disorder (GAD).

**Methods:**

A multicenter, prospective, double-blind, double-dummy, randomized controlled trial (RCT) was conducted at four hospitals in China. A total of one hundred thirty-three patients with GAD were enrolled from 2017 to 2019. This study aimed to evaluate the effects of a Traditional Chinese Medicine (TCM) JYW formula on GAD at eight weeks, with the use of Buspirone as the comparator. A stepwise dosing protocol was used (JYW: high dose 24 g/day, low dose 12 g/day; Buspirone: high dose 30 mg/day, low dose 15 mg/day) and the dose was adjusted depending on whether the treatment response of Hamilton Anxiety Scale (HAMA) score was less than or equal to 25% after one week. The primary outcome was a change in total score on the HAMA. The secondary outcomes included the Hamilton Depression Scale (HAMD), Clinical Global Impression (CGI) scale, and TCM Syndrome Scale. Adverse events were recorded using the Treatment Emergent Symptom Scale (TESS). Assessments were conducted at the baseline and 1, 2, 4, and 8 weeks.

**Results:**

A total of one hundred thirty-three participants were randomly assigned to the JYW group (*n* = 66) and the Buspirone group (*n* = 67). One hundred twenty-one patients (91%) completed at least one follow-up session. There were no significant differences between the two groups in terms of gender, age, disease course, HAMA, HAMD, CGI, and TCM Syndrome Scale scores at baseline (all *P* > 0.05). Repeated-measures analysis of variance revealed statistically significant time effects for the HAMA (*P*=0.002), HAMD (*P* = 0.018), and CGI (*P*=0.001) in both groups. Sensitivity analyses supported the credibility of the main results (*P* > 0.05). The group effect was not significant for the HAMA (*P*=0.43), HAMD (*P*=0.27), CGI (*P*=0.37), and TCM Syndrome Scale (*P*=0.86). Furthermore, there were no significant interaction effects between time and group in terms of the HAMA (*P*=0.47), HAMD (*P*=0.79), CGI (*P*=0.67), and TCM Syndrome Scale (*P*=0.69). After one week, 53 patients (80%) of the JYW group and 52 patients (78%) of the Buspirone group were adjusted to high doses. The interaction effect between time, group, and the dose was determined by repeated measures ANOVA test, and the HAMA score served as the outcome measure. The interaction effect between time and dose was statistically significant (*P*=0.04), which shows that high-dose JYW (24 g/day) was more effective in decreasing patients' HAMA scores than low-dose JYW (12 g/day), and Buspirone had the same effect, which means that high-dose Buspirone (30 mg/day) was more effective than low dose. (15 mg/day).

**Conclusions:**

The conclusion of this study supports that JYW and Buspirone can effectively alleviate the anxiety symptoms of GAD patients, which are both effective and safe for treatment of mild to moderate GAD. Besides, high-dose JYW or Buspirone are more effective than low-dose, which is of great importance in assisting clinical medication choice.

## 1. Introduction

Generalized anxiety disorder (GAD) is a prevalent mental disease characterized by uncontrollable and excessive apprehension that persists in any environmental circumstance. Dominant symptoms include persistent nervousness, trembling, muscular tension, sweating, lightheadedness, palpitations, dizziness, and epigastric discomfort. [1] Epidemiological data indicate that the lifetime prevalence of GAD is 3.7–11%, and the 12-month prevalence rate is 1.8% [2–4]. GAD is a chronic, disabling, complex psychiatric disease that has a low rate of full remission, [4, 5] often having profound effects on quality of life and social function [6] and places a heavy financial and psychological burden on families and society [7, 8].

Available therapies for GAD include pharmacotherapy, psychotherapy, and combined treatment approaches. Prior to the 1980s, benzodiazepines represented the most prescribed medication for GAD. However, benzodiazepines are for short-term use only because of their adverse side effects (e.g., sedation, withdrawal symptoms). Antidepressant medicines such as sertraline (a selective serotonin reuptake inhibitor, SSRI) or venlafaxine (a serotonin-norepinephrine reuptake inhibitor, SNRI) are recommended when the symptoms of GAD cause significant functional impairment. However, their side effects may limit clinical applications. Buspirone is a non-benzodiazepine anxiolytic and 5-hydroxytryptamine (5-HT) 1A receptor agonist approved for treating GAD in adults, with fewer sedation or withdrawal symptoms. Its effectiveness and tolerance have been demonstrated in multiple, double-blind, placebo-controlled trials. However, it is not the preferred first-line therapy for GAD due to its delayed onset of action [9–11]. Therefore, it is necessary to explore other appropriate and safe therapeutic approaches for the treatment of GAD.

Traditional Chinese medicine (TCM), a treatment modality that has been used in China for several thousand years, has attracted increasing attention in anxiety therapy [12]. GAD belongs to the category of “visceral impatience” in TCM. The main causes are six excessive pathogenic factors: internal injury due to seven emotions, lingering illness, yin-yang disharmony, flaring of heart fire, damage of nutrient qi and yin fluid, and uneasiness affecting the spirit. The *Jie Yu Wan* (JYW), a Chinese patent medicine, was derived from the Gan Mai Da Zao decoction and Xiao Yao San, the famous TCM formulated to treat depression and anxiety. The JYW formula was prepared in the form of concentrated pills and comprised of 10 medicinal compounds: Paeoniae Alba, Bupleuri, *Angelicasinensis*, turmeric, Poria, lily, *Albizia julibrissin*, licorice, light wheat, and jujube. This medicine is approved by the China Food and Drug Administration (CFDA) for treating depression and anxiety with the CFDA ratification number of GuoYaoZhunZi-B20020101. Previous studies have shown that the antidepressant therapeutic actions of JYW are potentially mediated through the adjustment of 5-HT and noradrenaline (NE) levels and the attenuation of the monoamine oxidase activity of multiple brain areas including hypothalamus, hippocampus, and prefrontal cortex. Besides, JYW exhibited anxiolytic effects by increasing the time percentage in the elevated plus-maze and has no significant inhibitory effects on the central nervous system [13, 14]. Finally, previous clinical studies have also proved that JYW has shown remarkable efficacy on GAD with few side effects [15]. The abovementioned findings suggest that JYW may have a therapeutic effect on GAD. Thus, the primary aim of this study was to assess the efficacy and safety of JYW in the treatment of GAD, which may provide a potential clinical treatment option.

## 2. Materials and Methods

### 2.1. Study Design

This study was a randomized, prospective, double-blind, double-dummy, and multicenter clinical study. Five hospitals in China participated in this trial, and Beijing Anding Hospital is the leading unit. The participating hospitals included Tangshan Fifth Hospital, Zhumadian Mental Hospital, Hangzhou Seventh People's Hospital, and Xiamen Xian Yue Hospital. All experimental procedures and protocols were approved by the institutional ethics committee of the Beijing Anding Hospital ((2017)63-201772FS-2) and registered on the Chinese Clinical Trial Registry website (ChiCTR-IPR-17013058). Participants were recruited from outpatient and inpatient departments at each center. They were informed of the detailed information and potential risks of the study by verbal and written information. Patients provided written informed consent prior to participating in the study, and their personal information was treated as confidential by the study staff. Participants who met the inclusion criteria were randomly allocated to visit into a JYW group or a Buspirone group at a 1 : 1 ratio. A stepwise dosing protocol was used, the dose was adjusted depending on whether the treatment response of HAMA score was less than or equal to 25% after one week. Outcomes were measured at baseline, and weeks one, two, four, and eight.

### 2.2. Participants

All patients meeting the following criteria were eligible for inclusion in the study: (1) aged 18 to 65 years old; (2) met the International Classification of Disease-10 (ICD-10) criteria for a primary diagnosis of GAD; [16] (3) met the TCM diagnostic criteria for fire derived from the stagnation of Liver-QI, including emotional impatience, irritability, fullness in the chest and hypochondrium, dry mouth, bitter mouth, constipation, headache, conjunctival congestion, tinnitus, reddened tongue with yellow fur, and pulse number; (4) Hamilton Anxiety Scale (HAMA) score >14 points and ≤29 points at screening and baseline; and (5) informed consent form signed by the participant or legal guardian.

### 2.3. Exclusion Criteria

Patients who met any of the following criteria were excluded from the study: (1) presence of glaucoma, any serious internal disorder affecting vital organs (i.e., heart, liver, kidney, endocrine, acute blood diseases), or acute and chronic inflammatory disease (e.g., systemic lupus erythematosus, ulcerative colitis); (2) experiencing suicidal ideations, history of epilepsy (except for febrile convulsion in childhood), or alcohol or medicine dependence within the past year; (3) anxiety secondary to other mental or physical diseases; (4) pregnant or breastfeeding during the study period; (5) history of Buspirone allergy or other medicine allergies; (6) participated in any other investigational medicine studies or clinical trials within the past 30 days or having taken monoamine oxidase inhibitors or Buspirone in the past four weeks; (7) without a guardian or unable to follow the directions of the medication; and (8) HAMA score reduction rate >25% [17, 18] or Hamilton Depression Scale (HAMD)17 item score >7 between screening and baseline (3–7 days).

### 2.4. Randomization and Masking

A random allocation sequence was generated by a trained statistician using statistical analysis system (SAS) software (SAS Institute, Cary, NC). Participants who met the inclusion criteria were randomly allocated to visit into two groups (i.e., treatment or control group) at a 1 : 1 ratio. Each patient was assigned a bag used to distribute the medicines. It remained unchanged throughout the study. An individual not directly involved in the study carried out medicine blinding, labeling, and packaging. The blinding codes were preserved and protected separately by Beijing Anding Hospital and the manufacturing company. An emergency letter could only be opened in the case of a serious adverse event. Researchers responsible for screening and recruiting participants, dispensing medicines, and outcome evaluation were blinded to group allocation throughout the study. Statistical analyses were carried out by independent statisticians who were only aware of group codes and were blinded to group allocation. The treatment medicine and matching placebo had a similar appearance, weight, and taste to maximize participant blinding.

### 2.5. Interventions

As detailed above, study participants were assigned to one of two groups: a JYW group (JYW formula and Buspirone placebo) or a Buspirone group (Buspirone and JYW placebo). The JYW formula was prepared in the form of concentrated pills (total weight = 4 g) and comprised of 10 medicinal compounds: *Paeoniae Alba* (1.14 g), Bupleuri (0.86 g), *Angelica sinensis* (0.57 g), turmeric (0.57 g), Poria (0.69 g), lily (0.69 g), *Albizia julibrissin* (0.69 g), licorice (0.34 g), light wheat (0.86 g), and jujube (0.57 g). The JYW is a Chinese patent medicine that has been approved for marketing in China. The dosage of JYW was obtained from the historical records in ancient literature, which combined modern experimental techniques and multiple clinical trials, to define the clinically effective safe doses [13, 14]. The JYW formula and placebo were supplied by Tiansheng Taifeng Pharmaceutical Co., Ltd. (Lhasa, Tibet, China, GuoYaoZhunZi-B20020101). The Buspirone and placebo were supplied by Enhua Pharmaceutical Co., Ltd. (Xuzhou, Jiangsu Province, China).

**Figure 1:**
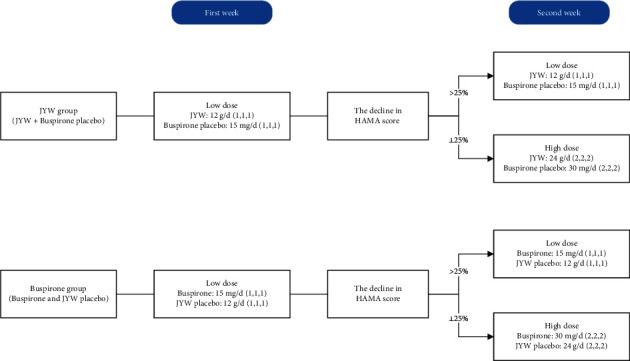
Detailed dose schedule. The number in () refers to the number of tablets taken in the morning, midday, and evening. 12 g/d, where “*d*” refers to day, not dose.

A stepwise dosing protocol was used in this study: Low dose-JYW formula or placebo (4 g, t.i.d.) with Buspirone or its placebo (5 mg, t.i.d.); and high dose-JYW formula or placebo (8 g, t.i.d.) with Buspirone or its placebo (10 mg, t.i.d) [19]. In the first week, patients were assigned a low dose. If the decline in HAMA score was ≤25% of the baseline score, [15] then a double dose or high dose was selected as the second-week dose. Otherwise, the low dose was used (Figure 1). The doses of JYW and Buspirone have been based on the medicine instructions and the pre-experimental study [20, 21].

Medicines were prepackaged to facilitate administration and placed into envelopes along with information about the visit period, drug number, instructions for use, and storage conditions. Pharmacists who were not involved in the study were responsible for dispensing the medicines. Participants were given detailed verbal and written instructions about how to take the medicine. They were also asked to return the previous treatment cycle's medication and receive the next treatment cycle's medication at the end of each visit. Treatment was continued for eight weeks unless a severe adverse event occurred or a participant withdrew from the study. Antipsychotics, strategic and systematic psychological therapy, or other physical therapies such as electroconvulsive therapy, MECT, transcranial magnetic stimulation, and transcranial direct current stimulation were not allowed. Patients were permitted to take prescribed medicines for their somatic disease which were recorded in detail.

### 2.6. Adverse Event and Serious Adverse Event

Adverse events (AE) were assessed at each visit. An AE was defined as any untoward medical occurrence in a participant which does not necessarily have a causal relationship with this study. All AE were considered into “possibly related,” “probably related”, or “definitely related.” According to the following criteria: (1) Possibly related: The occurrence of AE may be caused by the medicine. It cannot be determined whether the AE was caused by other factors, such as concomitant medications or concomitant diseases. The occurrence of AE was logically related to the timing of investigational medicine use, so a causal relationship between events and medicine use cannot be ruled out. (2) Probably related: The occurrence of AE may have been caused by the use of the investigational medicine. The timing of events is suggestive, such as adverse effects that subside after drug withdrawal. It is unlikely that other factors explain the phenomenon, such as concomitant medications or concomitant diseases. (3) Definitely related: The type of AE has been considered to be a side effect of the investigational drug and cannot be explained by other factors, such as concomitant medications and concomitant diseases. The timing of events strongly suggests a causal relationship, such as the response to withdrawal and administered again.

For the AE that occurred during the study period, the symptoms, degree, time of occurrence, duration, treatment measures, and procedures should be recorded in the case report forms (CRFs), and the correlation between the AE and the medicines should be evaluated. The occurrence of any of the following conditions should be regarded as a serious adverse event: hospitalization, prolonging hospital stay, permanent disability, affect work ability, life threatening emergencies or death, and cause congenital malformations, etc. All serious adverse events should be recorded in the serious adverse event report form.

AEs and SAEs were recorded at every study visit, using the treatment-emergent symptom scale (TESS). The researchers determined the correlation between adverse events and the experimental medicines.

## 3. Outcomes

Within 3–7 days screening period, all patients underwent a baseline evaluation, including demographic information (name, gender, age), medical history, physical and neurologic examination, and laboratory testing. Study day 1 was defined as the first day patients met enrollment criteria. Outcomes were measured at baseline and at weeks one, two, four, and eight.

The primary outcome was the change in HAMA score from baseline to 8 weeks. The HAMA is a 14-item diagnostic questionnaire used in the clinical and research setting to measure the severity of anxiety symptoms, involving two types of symptoms factors-psychic anxiety and somatic anxiety. The items are scored on a five-point scale (0 = not present to 4 = very severe). The total score is 0–56. It was divided into an absence of anxiety (0–6), possible anxiety (7–13), mild anxiety (14–20), moderate anxiety (21–28), and severe anxiety(29–56) [22]. The HAMA score has good reliability and acceptable validity to measure the severity of participants' anxiety [23, 24].

Secondary outcomes included changes in the HAMD, Clinical Global Impression (CGI) scale, and TCM Syndrome Scale. Depression severity was measured by using the 17-item HAMD scale. Evaluation criteria and methods were essentially identical to the HAMA's. The scale's seven-factor structure includes anxiety and somatization, weight, cognitive disorders, diurnal variation, block, sleep disorder, and hopelessness. [25] It has a high overall reliability as depressive outcome measure [26]. The CGI scale was used to assess the clinical efficacy of treatment. The scale was applicable across a broad range of psychiatric populations and consists of three items that assess the severity of illness, global improvement, and efficacy index [27]. The CGI has the characteristics of utility, sensitivity to change, and reliability in psychiatric research [28]. The TCM Syndrome Scale was developed by the Chinese medical team of experts. It contained over 90 items, assessing (n): mental symptoms (11), the head and face (7), the heart and chest (10), gastrointestinal symptoms (25), urologic symptoms (14), the reproduction system (10), and limb symptoms (11). In addition, two other TCM syndrome characteristics (i.e., tongue coating and pulse) are recorded as part of the scale. We used the two-round Delphi method to reach consensus. (See additional file for the process of the Delphi method). The evidence of reliability was conducted with the reliability coefficient of Cronbach's alpha (*α* = 0.935), which means that reliability of this questionnaire is high. The main safety parameters included routine blood tests (i.e., red blood cell (RBC), hemoglobin (HB), white blood cell (WBC), and platelet counts), routine urine tests (i.e., urine protein, urine sugar, WBC, RBC), a urine pregnancy test (when screening female patients), blood biochemistry tests (i.e., alanine aminotransferase (ALT), aspartate transaminase (AST), blood urea nitrogen (BUN), serum creatinine (Cr), and glucose and electrocardiograms (ECGs), which were carried out at baseline and the end of the 8 week intervention.

## 4. Statistical Analysis

Initially, the primary aim of the trial was to investigate the clinical efficacy of JYW in treating GAD, as stated in our protocol paper [19] and on ChiCTR-IPR. Base on the prior study, the response rate was 75% of Buspirone [29] and a response rate of 90% for JYW was assumed [15, 30, 31]. A prior power analysis was conducted with PASS Software version 15.0.5 (https://www.ncss.com/software/pass/), using an alpha of 0.05, a power of 0.8, and small to medium effect size (0.43) to determine the sample size. The sample size calculated was 83 for each group. Considering a dropout rate of 20%, a total of 200 participants were finally required.

Analyses and reporting were carried out in accordance with the Consolidated Standards of Reporting Trials (CONSORT) 2010 guidelines. [32](Table S1) CRF data were entered into EpiData 3.0 by two independent researchers in a double-blinded manner. Data were first examined for accuracy and consistency of data entry. A statistician blinded to the allocation of groups analyzed the data using IBM SPSS Statistics for Windows, version 20.0 (IBM Corp., Armonk, N.Y.). Continuous variables were presented as means ± standard deviations (SDs), while categorical variables were presented as frequencies or percentages. Baseline data were compared between the two groups using *t*-tests (normally distributed data), nonparametric tests (non-normally distributed data), and Chi-square tests. Repeated-measures analysis of variance (ANOVA) was used to evaluate primary and secondary outcomes. The significance of differences between the two methods was evaluated by noninferiority tests. Sensitivity analysis is mainly used to evaluate the robustness of the primary outcomes. Sensitivity analysis was conducted using *t*-test with four-week HAMA scores as the dependent variable and time as the independent variable. [33]The efficacy analysis was performed according to the intention-to-treat principle (ITT) and conducted on the Full Analysis Set (FAS), which comprised of all randomized participants. The main analysis data set comprised all randomized participants that took their assigned drug on at least one occasion. Missing data were imputed by using the Last-Observation-Carried-Forward (LOCF) method. Statistical significance was defined as *P* < 0.05.

## 5. Results

### 5.1. Comparison of Baseline Data

**Figure 2:**
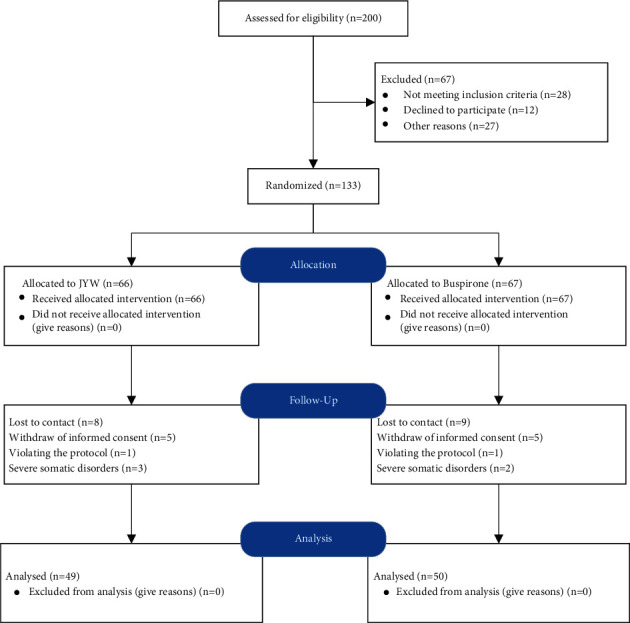
Study flowchart.

A total of 133 eligible patients were randomly allocated into a Buspirone (*n* = 67) and JYW (*n* = 66) group from January 1, 2017, to August 31, 2019. All patients completed the baseline assessment, 121 (91%) patients completed at least one follow-up evaluation, and 99 (75%) patients completed the 8-week treatment period. The proportions of patients attending each follow-up visit were similar in both groups. Data from 34 patients were imputed using the LOCF method (Figure 2).

Overall, 99 patients completed the study, with 49 in the JYW group and 50 in the Buspirone group. Participants were well matched for age, sex, race, and education level (Table 1). The mean (SD) age of patients was 46.25 years (SD = 11.57); the majority patients were female (67.42%) and Han race (97.72%). 75 (56.81%) patients with a duration less than 12 months, and 19(14.39%) patients with a duration more than 36 months. The mean HAMA score was 19.02 (SD = 3.98), with 89 (67.42%) patients scoring between 14–21 and 44 (33.33%) patients exceeding a score of 21 (Table 1).

### 5.2. Change of the HAMA Score in Two Groups

Due to attrition and missing data, 99 patients were included in the analysis of the primary outcome. The mean of 8-week HAMA scores were 6.67 (SD = 4.04) in patients allocated to the JYW group and 8.22 (SD = 4.63) in patients allocated to the Buspirone group. The change in HAMA scores of the two groups during the experimental period is shown in Table 2. Repeated-measures ANOVA revealed a significant time effect (*F* (1.76,170.37) = 300.36, *P*=0.000), but no significant group effect (*F* (1.00,97.00) = 0.78, *P*=0.38), and interactive effect between groups and time (*F* (1.76,170.37) = 0.94, *P*=0.38) (Table 2, model1).

Data from all participants were included in the efficacy analysis, according to the ITT principle, therefore, 133 participants were included in the FAS set analysis. The mean of 8-week HAMA scores were 9.21 (SD = 6.69) in patients allocated to the JYW group and 10.28 (SD = 4.68) in patients allocated to the Buspirone group (Table 2, model 2). We found that the results were consistent.

Sensitivity analyses were performed using *t*-test analysis to assess the stability of the results, using four-week HAMA scores as the dependent variable and time as the independent variable. The results of this analysis confirmed the results of the primary analysis (Table 2, models 3).

Patients who had less than 25% reduction in HAMA scores on day 7 were adjusted to high dose according to the study protocol (Figure 1). 53 patients (80%) of the JYW group and 52 patients (78%) of the Buspirone group were adjusted to high dose after one week. There was no statistically significant difference in the rate of attrition between the groups at baseline. The interaction effects between time, group, and dose were analyzed by multivariate analyses, using time as within-subjects factor, and dose and group as between-subjects factors. There was a significant time effect (*F* (1.71,195.10) = 12.02, *P* < 0.001), and the interaction effect between time and dose was statistically significant (*F* (1.71,195.10) = 3.38, *P*=0.04), showing that high-dose had a significant better compared to low dose. However, no significant differences were found between JYW and Buspirone (*F* (1.71,195.10) = 1.34, *P*=0.26).

Noninferiority analysis was conducted to verify the efficacy of JYW in treatment of GAD was not inferior to Buspirone. According to the previous studies and the guidance method of noninferiority test, the noninferiority margin of Buspirone was 5.7 [34, 35]. The score of JYW group remained unchanged, and the *t*-test was carried out (*P* < 0.001). The result indicated that JYW was noninferior to Buspirone (Table 2, model 4).

**Figure 3:**
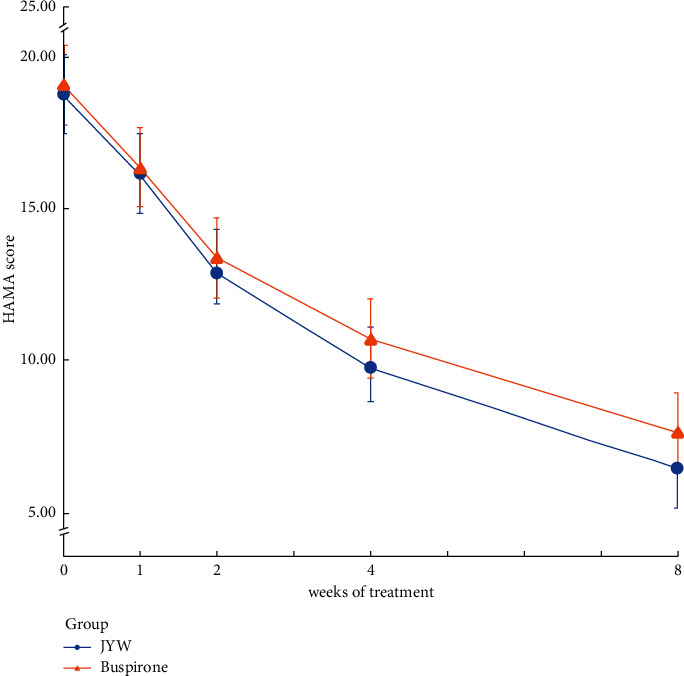
Changes in score from baseline on HAMA over time. The time trend effect was estimated using a repeated-measures ANOVA model, with time as the independent variable. The abscissa axis indicates weeks of treatment. The baseline, 1,2,4, and 8 weeks' time points are displayed. The ordinate shows the mean HAMA scores of every time point.

The time trend effect was estimated by using a repeated-measures ANOVA model, with time as the independent variable. The plot clearly showed a decreasing time trend effect in both two groups. Although the decrease in the HAMA score in the JYW group during the intervention was greater than in the Buspirone group, the two groups showed no evidence of interaction effect (Figure 3).

### 5.3. Change of the HAMD, CGI, and TCM Syndrome Score in Two Groups

Table 3 displayed the index change in the two groups before and after treatment, involving HAMD, CGI, and TCM syndrome. The adjusted proportional difference in HAMD scores across eight weeks was 3.14 (95% CI = 2.73–3.54). After eight weeks of treatment, there was a significant time effect in HAMD scores (*F* (2.16,282.51) = 90.79, *P* = 0.018]. We observed the similar results for the CGI. Specifically, the adjusted proportional difference in CGI scores across four weeks was 2.23 (95% CI = 2.09–2.36) and a significant time effect in CGI scores (*F* (1.96,256.82) = 146.16, *P*= 0.001). Differences in TCM Syndrome Scale scores did change over time (*F* (1.71,224.54) = 156.81, *P* = 0.001). There was no group effect for HAMD (*F* (1.00,131.00) = 0.71, *P* = 0.27), CGI (*F* (1.00,131.00) = 0.26, *P* = 0.37), and TCM Syndrome Scale (*F* (1.00,131.00) = 0.01, *P* = 0.86). We did not observe any significant interaction effects between time and group for HAMD (*F* (2.16, 282.51) = 0.22, *P* = 0.79), CGI (*F* (1.96,256.82) = 0.31, *P* = 0.67), or TCM Syndrome Scale (*F* (1.71,224.54) = 0.65, *P* = 0.69) (Table 3).

### 5.4. Comparison of the Incidence of Adverse Reactions between Two Groups

Based on the Council for the International Organization of Medical Sciences (CIOMS), adverse events with a frequency ≥10% were classified as very common and with a frequency ≥1% but <10% were classified as common. 5 very common adverse reactions occurred in the Buspirone group: dry mouth (18%), insomnia (14%), constipation (12%), weight gain (12%), skin symptoms (12%). Insomnia (12%) was the only very common adverse reaction that occurred in the JYW group. 14 common adverse reactions occurred in the Buspirone group. Specifically, the incidence rate of nasal congestion, sweat, nausea, and vomiting, diarrhea, tachycardia, loss of weight, and headache was 2%; the incidence rate of excitement, a hematological abnormality, myotonia, and anorexia was 4%; the incidence rate of sleep and blurred vision was 6%, and the incidence rate of liver function was 8%. 15 common adverse reactions occurred in the JYW group. Specifically, the incidence rate of excitement, a hematological abnormality, tremor, dry mouth, nausea and vomiting, diarrhea, hypotension, weight gain, skin symptoms, and headache was 2%; the incidence rate of tremors hypokinesis, liver function, constipation, and increased saliva was 4%; and the incidence rate of depression was 6%. A chi-squared test was used to analyze the TESS results. The difference between the two groups was statistically significant at eight weeks (*P* = 0.021), which means that the side effect of JYW was less than the Buspirone. The most obvious are the nervous system (*P* = 0.02) and the cardiovascular system (*P* = 0.047), which showed that the adverse reactions of the nervous system and the cardiovascular system in JYW group were mild. Systems incidence rate of adverse reactions in the JYW group was significantly lower compared with that in the Buspirone group (Table S2).

## 6. Discussion

To the best of our knowledge, this was the first randomized controlled trial (RCT) that compared the effectiveness of JYW and Buspirone for the treatment of GAD. The majority of enrolled patients presented with mild to moderate GAD. Outcomes included HAMA, HAMD, CGI, and TCM syndrome. The results indicated that the antianxiety effects of JYW was noninferior to Buspirone. The HAMA score of the JYW group decreased over time (*P* < 0.001), which to be noninferior to Buspirone group at weeks 1, 2, 4, and 8 post-treatments. There were no significant differences between the two groups (*P* > 0.05). In addition, efficacy findings confirmed that high-dose JYW (24 g/day) had a higher significant of decrease patients' HAMA scores than low-dose JYW (12 g/day) (*P* < 0.05) and Buspirone had the same effect, which means that high-dose (30 mg/day) had a better effect than low-dose (15 mg/day) (*P* < 0.05). Our results showed that JYW was a new treatment approach that could improve mental health and physical symptoms in patients with mild to moderate GAD.

Findings from this trial demonstrate that JYW was not inferior to Buspirone with regard to clinical anti-anxiety efficacy in context, both are effective regimen for treatment of GAD. However, in terms of side effects, the cardiovascular and nervous system side effects in the JYW were relatively mild, which is expected to be more easily accepted by patients with GAD. Previous literature studies reported that the Buspirone may lead to the malignant syndrome [36], but no data were reported for JYW. Buspirone is a partial 5-HT1A receptor agonist. The mechanism of action for Buspirone is well-characterized, which has a strong affinity for serotonin 5HT1a receptors [33]. In contrast, due to the variety components of JYW, the detailed mechanism behand JYW needs further investigation.

According to neurobiological studies, GAD may be associated with declines in cerebral blood flow and metabolic function, neuroanatomical impairments involving regions such as the amygdala and dorsolateral prefrontal cortex, neurotransmitter abnormalities, and altered adrenergic function. [37–40] Further, the immune-mediated inflammatory disease may induce the hippocampus, hypothalamus, medulla oblongata, brain stem, and other parts of the brain to dysregulate the release of histamine, serotonin, and other neurotransmitters, leading to anxiety or depression [41]. Another study mentioned that the level of inflammatory cytokines such as Interleukin-1 (IL-1), IL-6, tumor necrosis factor (TNF)-*α*, and C-reactive protein (CRP) in patients with GAD is different from the general population. [42] It affects the pathophysiological process and plays an important role in the immune regulatory response of GAD [43].

JYW is fabricated from the Xiao Yao San (XYS) and Gan Mai Da Zao (GMDZ) decoction. Preclinical studies have been conducted with these components in order to understand the potential mechanisms of action of XYS and GMDZ. First, animal experiments have demonstrated the potent antianxiety effect of XYS and GMDZ. Specifically, XYS improves anxiety or depressive-like behavior by modulating gut microbiota and immune function, regulating the Apelin-APJ System in the hypothalamus, and the structure of related brain regions. [44–47] The GMDZ decoction possesses anxiolytic-like effects in elevated plus-maze, light/dark box, and open field tests in mice, which are similar to those observed with diazepam and Buspirone. The mechanism of action may be related to the regulation of 5-HT, NE, the hypothalamic-pituitary-adrenal (HPA) axis, and *γ*-aminobutyric acid _A_ (GABA _A_) receptors [48, 49]. Second, JYW contains complex chemical compositions. Several studies of the pharmacological mechanism of herbs, including both animal experiments and clinical trials, support the antianxiety effects. Thus, some pharmacological lines of reasoning may explain the effects of JYW. For example, Radix Bupleuri extracts include in the prescription exhibited various biological activities (e.g., anti-inflammatory, neuroprotective, and immunomodulatory effects) [50] that can ameliorate depression symptoms by increasing serum levels of Nerve Growth Factor (NGF) and Brain-Derived Neurotrophic Factor (BDNF) [51]. The Radix Bupleuri and Radix Paeoniae Alba drug pair can significantly elevate the concentrations of 5-HT and NE in the hippocampal and cortical tissues [52, 53]. Several studies have found that extracts from the fractions of *Angelica keiskei*, the active constituent of *Curcuma longa*, and the flavonoid-rich ethanol extracts of licorice root have potential sedative and anxiolytic effects [54–56]. Various studies of Poria Coco's fungus have demonstrated its marked anti-inflammatory activity in different experimental models of acute and chronic inflammation [57]. Third, GAD belongs to the category of “visceral impatience” in TCM. The main causes are six excessive pathogenic factors including internal injury due to seven emotions, lingering illness, yin-yang disharmony, flaring of heart fire, damage of nutrient qi and yin fluid, and uneasiness affecting the spirit. Fire derived from the stagnation of Liver-QI is the most common type of GAD [58]. XYS and GMDZ decoction is a classic TCM prescription, first written by Zhang Zhong-jin, commonly used for the treatment of depression and lily disease. It should be noted that the clinical manifestations of depression or lily disease recorded by ancient texts were very similar to that of GAD (e.g., fidgeting and irritability, sleep disorder, headache and dizziness, expansion, fullness in both flanks). Previous research also has confirmed that these two prescriptions were safe and might effectively improve anxiety and TCM symptoms in patients with GAD, and improving sleep quality and quality of life [59, 60].

This study has several limitations. First, although several studies have suggested that TCM can regulate inflammatory responses, [61, 62] it is unclear whether JYW has a similar function. This study was conducted as a clinical investigation, we only used scale to evaluate reductions in anxiety symptoms. Further investigation in the biological mechanism of action is needed. Second, TCM contains complex chemical compositions. Thus, the pharmacological action of antianxiety prescriptions requires further research by using a multicomponent approach. Although animal experiments have confirmed that individual medicines in the prescription have potential antianxiety mechanisms, [49, 52] it is unknown whether JYW has the same mechanism of action. This needs to be explored further. Third, there were no differences between JYW and Buspirone in improvements in TCM syndrome. This contrasts with the findings of previous studies [59, 63] and might be a result of varying statistical methods and follow-up periods. Fourth, we were unable to obtain reliable efficiency rates since there were no prior RCTs of JYW for GAD. Thus, we estimate the required sample size was 200. Due to time constraints, the study was terminated prior to reaching this targeted sample size. Fifth, there was a relatively high dropout rate, including 10 participants who revoked consent, 2 participants who did not follow the study protocol, and 22 participants lost to follow-up for many reasons, such as the inconvenience and the longer distance, the insufficient efficacy, or patient recovered well and no require long treatment. Last, this research was conducted according to the prespecified protocol, and we followed the protocol thoroughly. According to the instructions, the Buspirone starting and recommended dose was 10–15 mg/d on the first week, the dose would be increased to 20–30 mg/d for the second week of the treatment period. Although many studies have shown that TCM has antidepressant or antianxiety effects [14, 46, 49], high dosages of TCM may be to achieve the same effects like western medicine in clinical practice. In order to raise the completion rates in the trial and avoid the dropout, we designed the adjustment time of the drug to be 1 week according to the clinical experience, not 2 weeks or 4 weeks. This problem should be considered and improve the therapeutic strategies when designing future trials.

Considering the abovementioned limitations, future research will be able to consider the following. For example, we plan to continue to recruit patients to increase our sample size in future studies. Inflammatory factors such as IL-6 and TNF are important regulatory factors in the development of GAD. It remains to be investigated whether JYW could relieve anxiety by regulating the inflammatory immune processes. Relevant research has been underway. Lastly, many omics technologies, such as transcriptomics, proteomics, and metabolomics, have been widely applied in medical research. They are potential tools to better understand the pathogenesis of GAD, the mechanisms of medicines, and uncovering more favorable approaches in TCM therapy. We will apply proteomics to further investigate the detailed mechanism of JYW action and the relationship between the dose and efficacy in future study.

## 7. Conclusions

The main conclusion of this study was that JYW and Buspirone can effectively alleviate the anxiety symptoms of GAD patients, which are both effective and safe for treatment of mild to moderate GAD. Not only that, high-dose JYW or Buspirone are more effective than low dose, which is of great importance in assisting clinical medication choice.

## Figures and Tables

**Table 1:** Demographic and baseline data (*x* ± *s*).

	JYW (*n* = 66), *n* (%)	Buspirone (*n* = 67), *n* (%)	*t/x* ^2^	*P*
Age	46.96 ± 12.31	45.55 ± 10.84	0.70	0.49
*<30*	8 (12.12%)	5 (7.5%)		
*30–50*	28 (42.42%)	35 (52.23%)		
*>50*	29 (43.94%)	27 (40.29%)		
*Gender*				
*Male*	22 (33.33%)	21 (31.34%)	1.03	0.60
*Female*	44 (66.67%)	46 (68.57%)		
*Race*				
*Han*	65 (98.48%)	64 (95.52%)	−0.58	0.56
*Minorities*	1 (1.5%)	2 (3%)		
*Educational level*				
*Primary education or less*	6 (9.1%)	8 (12.94%)	0.33	0.74
*Secondary education*	32 (48.48%)	34 (50.75%)		
*Tertiary education*	28 (42.42%)	25 (37.31%)		
Duration	0.5 (0,1.85)	0.67 (0,2.08)	−0.71	0.48
≤12 months	36 (54.55%)	36 (53.73%)		
12 < months < 25	16 (24.24%)	15 (22.39%)		
25 < months < 36	6 (9.1%)	5 (7.5%)		
>36 months	8 (12.12%)	11 (16.42%)		
Baseline HAMA^a^	18.95 (4.07)	19.09 ± 3.92	−0.57	0.57
14 ≤ HAMA ≤ 21	51 (77.27)	47 (70.15)		
21 ＜ HAMA ≤ 29	15 (22.73)	20 (29.85)		
Baseline HAMD^a^	6.79 ± 1.26	6.87 ± 1.39	−0.41	0.68
Baseline CGI^a^	3.73 ± 0.97	3.80 ± 0.83	−0.77	0.67
Baseline TCM syndrome^a^	102.42 ± 38.12	99.52 ± 42.39	0.50	0.62

^a^Data are presented as *n* (%) or mean (SD). Duration: the disease duration of GAD.

**Table 2:** Primary outcome analyses (the HAMA total score).

	*n*	JYW, mean (SD)	*n*	Buspirone, mean (SD)	*F*	*P*
Model 1	49		50			
Baseline		18.8 (3.73)		19.14 (3.66)		
1 week		16.31 (3.60)		16.58 (3.83)		
2 weeks		13.37 (4.00)		13.72 (4.82)		
4 weeks		10.31 (4.56)		11.06 (5.02)		
8 weeks		6.67 (4.00)		8.14 (4.62)		
Time effect		…		…	300.36	0.001
Group effect		…		…	0.78	0.38
Group*∗*time^a^		…		…	0.78	0.38
Model 2	66		67			
baseline		18.95 (4.07)		19.09 (3.92)		
1 week		16.65 (4.15)		16.58 (4.07)		
2 weeks		14.14 (4.72)		14.85 (5.31)		
4 weeks		11.91 (6.09)		12.58 (6.02)		
8 weeks		9.21 (6.70)		10.28 (4.68)		
Time effect		…		…	193.54	0.001
Group effect		…		…	0.55	0.46
Group*∗*time^a^		…		…	0.49	0.57
Model 3	53	10.79 (5.60)	54	11.00 (5.15)	−0.20	0.84
Model 4	53	10.79 (5.59)	54	5.3 (5.15)	5.27	0.001

Data are presented as mean (SD). The adjusted proportional difference can be interpreted as the difference in scores between the randomized groups, expressed as a proportion (or percentage). Model 1: primary analysis model, includes data for participants who completed the entire study (*n* = 99). Model 2: ITT analysis conducted on the FAS (*n* = 133). Model 3: sensitivity analyses (*n* = 108). Model 4: noninferiority test and analysis (*n* = 108). ^a^Interaction effect between group and time.

**Table 3:** Repeated measures analyses of continuous secondary outcomes at weeks 1–8.

	JYW, mean (SD) (*n* = 66)	Buspirone, mean (SD) (*n* = 67)	*F*	*P*
*HAMD*				
Baseline	6.75 (0.20)	6.95 (0.20)		
1 week	5.88 (0.18)	6.12 (0.18)		
2 weeks	4.99 (0.23)	5.17 (0.23)		
4 weeks	3.91 (0.30)	4.46 (0.30)		
8 weeks	3.02 (0.29)	3.25 (0.29)		
Time effect	…	…	90.79	0.001
Group effect	…	…	0.71	0.40
Group*∗*time^a^	…	…	0.22	0.82
*CGI*				
Baseline	…	…		
1 week	3.42 (0.61)	3.45 (0.63)		
2 weeks	2.95 (0.62)	3.09 (0.85)		
4 weeks	2.56 (0.95)	2.60 (0.95)		
8 weeks	2.17 (1.13)	2.24 (1.14)		
Time effect	…	…	146.16	0.001
Group effect			0.26	0.61
Group*∗*time^a^	…	…	0.31	0.73
*TCM syndrome*				
Baseline	102.42 (38.12)	99.52 (42.39)		
1 week	89.94 (37.32)	89.43 (40.47)		
2 weeks	77.35 (37.65)	79.90 (42.81)		
4 weeks	67.53 (40.81)	68.60 (43.91)		
8 weeks	55.58 (42.83)	57.84 (44.72)		
Time effect	…	…	156.81	0.001
Group effect			0.01	0.94
Group*∗*time^a^	…	…	0.66	0.50

^a^Interaction effect between group and time.

## Data Availability

The data that support the conclusions of this study are available from the corresponding author Jia Hongxiao at any time upon reasonable request.

## Supplementary Materials

Supplementary Materials The process of the Delphi method, the CONSORT 2010 checklist (Table S1), and the result of adverse reaction at eight weeks (Table S2) are provided as additional file.
