# Supplementary material for: Effects of Jie Yu Wan on Generalized Anxiety Disorder: A Randomized Clinical Trial
Source: Evid Based Complement Alternat Med. 2022 Apr 8;2022:9951693. doi: 10.1155/2022/9951693 (PMC9012658; doi:10.1155/2022/9951693)
Supplement: Supplementary Materials — The process of the Delphi method, the CONSORT 2010 checklist (Table S1), and the result of adverse reaction at eight weeks (Table S2) are provided as additional file. [file 9951693.f1.zip › 9951693.f1/Process of Delphi method.docx]

Process of Delphi survey

The expert survey questionnaire was constructed based on a review of the relevant literature. The first-round questionnaire was designed by the combination of the entries screening and a structured questionnaire with an open-ended answer format. The second-round questionnaire was conducted based on the objective-scoring and the specific comments. The conditions of the experts and researchers of selection were as follows: (1) specialists in the field of psychosis areas; (2) a senior title is required; (3) ten years or more relevant work experience; (4) be able to complete several rounds of survey; (5) the regional distribution of experts. The first round was carried out in July 2017, experts were invited through email. A total of 20 experts accepted the invitation and 17 experts (85% response rate) responded to the invitation. The seconded round of the questionnaire was conducted in Oct 2017. All questionnaires were administered electronically. A total of 20 experts (100% response rate) responded to the invitation.

All experts selected the importance of the items independently based on their professional knowledge and clinical experience. There are five voting opinions of each item: 1) Agree completely; 2) Agree, but with some reservations; 3) Agree, but have great reservations; 4) Disagree but have reservations; 5) disagree completely. Items were considered if more than 80% of the experts chose 1) + 2). Eliminated the disagree completely entries and combined the contents are the same. There were 125 out of 235 selected items in the first-round Delphi survey, and 74 out of 125 items was selected as the final items in the second round of expert questionnaire.
