# Supplementary material for: Effects of Jie Yu Wan on Generalized Anxiety Disorder: A Randomized Clinical Trial
Source: Evid Based Complement Alternat Med. 2022 Apr 8;2022:9951693. doi: 10.1155/2022/9951693 (PMC9012658; doi:10.1155/2022/9951693)
Supplement: Supplementary Materials — The process of the Delphi method, the CONSORT 2010 checklist (Table S1), and the result of adverse reaction at eight weeks (Table S2) are provided as additional file. [file 9951693.f1.zip › 9951693.f1/Tabel S2.docx]

Table S1: Comparison of the adverse reactions in the two groups at eight weeks with TESS (n)

|  |  | JYW  (n=49） | Buspirone  （n=50） | *p* |
| --- | --- | --- | --- | --- |
| Total |  | 5 | 14 | 0.021 |
| Behavioral toxicity | Total | 1 | 5 | 0.05 |
|  | Toxic of consciousness |  |  |  |
|  | Excitable or intense |  | 2 |  |
|  | depression |  |  |  |
|  | Increase in activity |  |  |  |
|  | Decrease in activity | 1 |  |  |
|  | insomnia |  | 3 |  |
|  | somnolence |  | 1 |  |
| Assay | Total | 4 | 7 | 0.22 |
|  | Hematological abnormalities | 1 | 4 |  |
|  | abnormal liver function | 2 | 4 |  |
|  | Abnormalities in urine | 1 |  |  |
| Nervous system | Total | 1 | 5 | 0.05 |
|  | myotonia |  |  |  |
|  | tremors |  |  |  |
|  | torsade action |  |  |  |
|  | akathisia |  |  |  |
|  | dry mouth | 1 | 5 |  |
| Autonomic nervous system | Total | 0 | 4 | 0.02 |
|  | nasal congestion |  | 1 |  |
|  | Blurred vision |  |  |  |
|  | Increase of saliva |  | 3 |  |
|  | Sweating |  |  |  |
|  | Nausea and vomiting |  | 2 |  |
|  | diarrhea |  | 1 |  |
| Cardiovascular System | Total | 1 | 5 | 0.047 |
|  | hypotensive |  |  |  |
|  | fainting |  |  |  |
|  | tachycardic |  |  |  |
|  | hypertension |  | 2 |  |
|  | ECG abnormalities | 1 | 3 |  |
| Others | Total | 0 | 2 | 0.08 |
|  | skin symptoms |  |  |  |
|  | weight gain |  |  |  |
|  | weight loss |  | 1 |  |
|  | appetite loss or anorexia |  |  |  |
|  | headache |  | 1 |  |
|  | tardive dyskinesia |  |  |  |
|  | others |  |  |  |
